# Coherent creation and destruction of orbital wavepackets in Si:P with electrical and optical read-out

**DOI:** 10.1038/ncomms7549

**Published:** 2015-03-20

**Authors:** K.L. Litvinenko, E.T. Bowyer, P.T. Greenland, N. Stavrias, Juerong Li, R. Gwilliam, B.J. Villis, G. Matmon, M.L.Y. Pang, B. Redlich, A.F.G. van der Meer, C.R. Pidgeon, G. Aeppli, B.N. Murdin

**Affiliations:** 1 Advanced Technology Institute and SEPNet, University of Surrey, Guildford GU2 7XH, UK; 2 London Centre for Nanotechnology and Department of Physics and Astronomy, University College London, London WC1H 0AH, UK; 3 Radboud University, Institute for Molecules and Materials, FELIX Laboratory, Toernooiveld 7c, 6525 ED Nijmegen, The Netherlands; 4 Institute of Photonics and Quantum Sciences, SUPA, Heriot-Watt University, Edinburgh EH14 4AS, UK; 5 Departments of Physics, ETH Zurich and EPF Lausanne and Paul Scherrer Institute, 5232 Villigen, Switzerland

## Abstract

The ability to control dynamics of quantum states by optical interference, and subsequent electrical read-out, is crucial for solid state quantum technologies. Ramsey interference has been successfully observed for spins in silicon and nitrogen vacancy centres in diamond, and for orbital motion in InAs quantum dots. Here we demonstrate terahertz optical excitation, manipulation and destruction via Ramsey interference of orbital wavepackets in Si:P with electrical read-out. We show milliradian control over the wavefunction phase for the two-level system formed by the 1*s* and 2*p* states. The results have been verified by all-optical echo detection methods, sensitive only to coherent excitations in the sample. The experiments open a route to exploitation of donors in silicon for atom trap physics, with concomitant potential for quantum computing schemes, which rely on orbital superpositions to, for example, gate the magnetic exchange interactions between impurities.

## Abstract (web summary)

Impurity spins in silicon can be controlled with microwaves and then read-out electrically, offering a promising platform for quantum information applications. Here, the authors show that terahertz pulses can be used to address the orbital degree of freedom as well, which can also be detected electrically.

## Graphical abstract

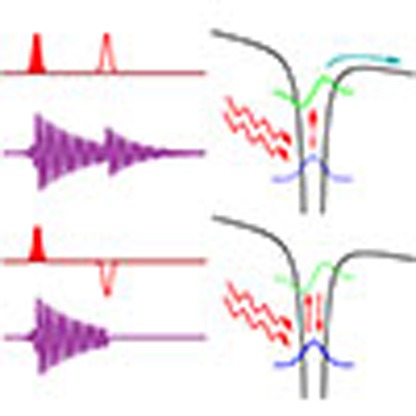

Control of quantum states has already led to many important technologies from pulsed nuclear magnetic resonance to optical lasers. While the early experiments by Rabi *et al*. have remained influential in atomic physics for the entire period since they were originally performed, their significance has become much greater to engineering physics over the last two decades in the context of the development of quantum information technology. One of the key experiments was that of Ramsey that showed how to use pulsed radiation to measure atomic excitation energies with high resolution^1^, and at the same time also demonstrated control over the wavefunction of the same two-level atomic systems, in the form of the much-celebrated ‘Ramsey fringes’. A second development has been the electrical read-out of atomic states in semiconductors^2^,^3^, recently focusing especially on quantum-controlled spins and charges in silicon and GaAs^4^,^5^,^6^,^7^,^8^,^9^,^10^,^11^,^12^. Here, we take advantage of coherent, tuneable pulsed THz radiation from a free-electron laser (FEL)^12^,^13^,^14^,^15^,^16^,^17^ to demonstrate electrical read-out of coherent orbital excitations for the phosphorous donor in silicon.

Beyond underpinning contemporary information technology, silicon is an excellent substrate for quantum information science^9^,^10^,^11^ partly because of the enormous potential advantage that a large number of atoms can be lithographically deposited with atomic resolution^18^,^19^. Most of the attention has been focussed on impurity spins, for which microwave control and electrical read-out have been demonstrated^6^,^7^, with the longest decoherence times for any solid-state system^8^. There has been much less exploration of the orbital degree of freedom because, given the low Rydberg energy in the heavily screened silicon ‘vacuum’^2^,^20^, terahertz (THz) technology is a prerequisite. The availability of free-electron lasers has opened this topic, allowing demonstration of incoherent relaxation^13^,^14^,^17^ and photon echoes^15^, corresponding to T_1_ and T_2_ processes for orbital transitions of impurities in silicon. Solid-state THz sources have also been used for field ionization studies^22^.

In this work, we advance silicon orbitronics by demonstrating electrical detection of Ramsey fringes for the 1*s*→2*p* transition of Si:P ensembles containing as few as 10^5^ donors. The fringes, manifestations of the desired wavepacket creation and destruction, are easy to establish in the THz regime—especially compared with the visible^21^, because in the THz domain free space optics and ordinary mechanical delay stages readily provide sub-wavelength resolution. From the decay of the fringes, we obtain T_2_*, the inhomogeneous decoherence time, and we show that the electrically detected result is consistent with the T_2_* obtained via all-optical echo detection after two Ramsey pulses. Apart from showing that a simple silicon wafer with some electrical contacts is sufficient to perform one of the classic experiments of atomic physics, our measurements should lead to future implementations of quantum gates.

## Results

### Electrically detected Ramsey fringes

**Figure 1:**
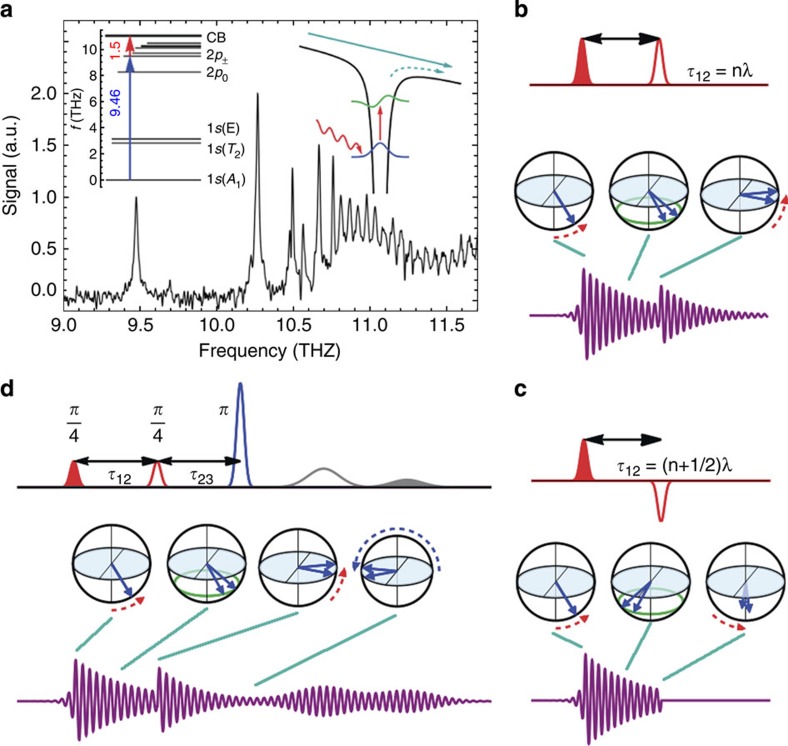
Level scheme and Ramsey interference. (**a**) The main figure shows the photothermal ionization spectrum (PTIS—black trace) of the Rydberg transitions for Si:P. Top left inset: the Rydberg state energies below the ionization threshold with the transition frequency for 1*s*(*A*_1_)→2*p*_±_ transition shown in THz (blue arrow), along with the phonon energy required for ionization of the excited state (red arrow). Top right inset: schematic of the potential for the phosphorous impurity in Si under external bias, along with the 1*s* and 2*p* wavefunctions. The curly red line represents the incoming photon that leaves atoms excited in the 2*p*_±_ state, and phonons collapse (dashed blue line) the wavefunction. A (small) fraction is ionized that is proportional to the probability for the excited state, and the ionization is detected in the conductivity. (**b**) The Ramsey sequence showing constructive interference (with π/4 pulses by way of example for consistency with **d**). The pulse sequence is shown at the top, and the *y*-component of the Bloch vector (laboratory frame), proportional to the polarization, is at bottom. (**c**) Same as for **b** for an extra half cycle delay and consequent destructive interference. (**d**) A Ramsey sequence followed by a rephasing pulse for optical echo read-out.

Figure 1a shows a schematic of the potential for a phosphorous impurity in silicon (Si:P) under applied bias, along with the 1*s* and 2*p* wavefunctions, and the bound Rydberg state energies below the ionization threshold. The electrical detection mechanism^2^,^3^, termed photothermal ionization spectroscopy (PTIS), is based on the much higher thermal ionization probability for an excited 2*p* state than the ground 1s state (see again Fig. 1a), implying that the sample conductivity will be enhanced even after non-ionizing photoexcitation from the 1*s* to 2*p* states. The main part of Fig. 1a is the resulting photoconductivity spectrum under small-signal illumination, showing resonances corresponding to transitions between the bound states. Previous workers have taken advantage of PTIS for continuous wave spectroscopy in the small-signal, linear regime^2^, or even incoherent pulsed dynamics^3^, but here we exploit it for coherent, time domain experiments.

The free-electron laser ‘FELIX’ is ideally suited as a pump for coherent manipulation of the donor orbitals in silicon because it is tuneable over the required THz (far infrared) range. Ti:sapphire-based THz transient systems are also useful for dynamics^22^, but have very wide bandwidth and are not appropriate for resonance experiments such as these. FELIX provides transform-limited pulses with controllable duration^16^, high peak pulse energy and high polarization purity, for use in pulsed experiments.

To visualize the coherent, pulsed excitation of a two-level quantum oscillator such as that defined by the 1*s* and 2*p* levels of a hydrogenic atom, one often makes use of the Bloch sphere (Fig. 1b–d), which represents the amplitude and relative phase of the coefficients in the superposition. In this picture, a vector pointing towards either of the poles represents the system in one of the eigenstates (we shall use the south pole to be the ground state, and the north pole to be the excited state), while a 50:50 superposition is represented by an equatorial vector and the phase is given by the azimuthal angle. After coherent, resonant excitation by a laser pulse, the Bloch vector is left with a rotation about the *x* axis by an angle, *θ*, proportional to the pulse area (the product of the pulse electric field amplitude and its duration), and in the dark the vector rotates about the *z* axis with the natural frequency (NB: in this picture we do not use a co-rotating reference frame). The Ramsey sequence (Fig. 1b) consists of a pair of equal pulses. The first Ramsey pulse leaves the Bloch vector rotated by *θ* and the effect of the second pulse depends on its precise arrival time. If it arrives after a whole number of periods of the natural oscillation, the light constructively interferes with the electronic state producing a final elevation of 2*θ*. If a half-integer number of periods has elapsed, the light–matter interference is destructive and the final elevation is zero. Inhomogeneous broadening is produced by the random arrangement of the different isotopes of silicon around each phosphorus impurity, which means that the oscillators all have slightly different natural frequencies. The primary effect of this broadening is that Bloch vectors precess at different rates, which means that there is a spread of phases when the second Ramsey pulse arrives and the interference contrast decays. Conventionally, π/2 pulses are used for Ramsey spectroscopy, but any equal area pulses produce the same fringes and π/4 pulses are convenient for echo detection experiments described later.

Mixtures of 1*s* and 2*p* states produced by coherent THz pulse sequences, such as Ramsey pairs illustrated in Fig. 1b,c, can be detected in the electrical conductivity, which is sensitive primarily to the 2*p* occupancy via the subsequent, much-longer lived, ionization. Our method allows us to deduce coherent control of the two-level system because the rectifying nature of electrical detection implies that for given coherent oscillations in the hydrogen-like states of the silicon impurities at given pulse delays, the photocurrent signal allows for read-out of their net amplitude. Said another way, the amplitude of the coherent wave superposition will affect the probability of thermally assisted photoionization.

**Figure 2:**
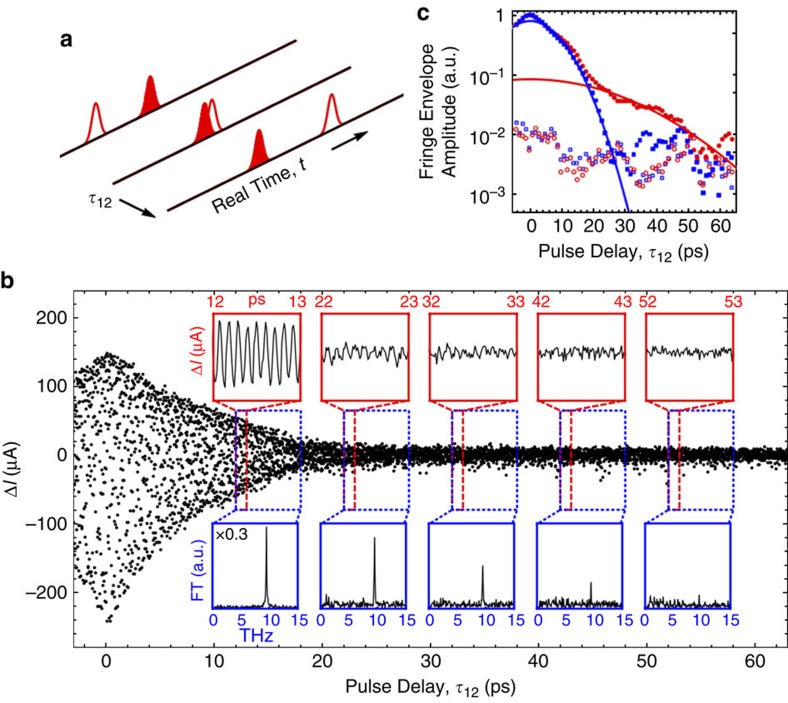
Electrically detected Ramsey interference. (**a**) The pulse sequences, showing pump pulses 1 (solid) and 2 (open), with time delay *τ*_12_ between them. (**b**) The experimental integrated photoconductivity signal—with the laser tuned to 9.46 THz corresponding to the 1*s*(*A*_1_)→2*p*_±_ transition—as a function of time delay between the pump pulses. The top row of insets shows the oscillations in expanded view. The bottom row of insets shows Fourier transforms (FTs) of the sections. (**c**) The magnitude of the envelope of the fringes in (**b**) data (red), produced with an FT filter centred on the laser frequency (solid symbols) and a FT filter set off-resonance to indicate the noise level, which is not Gaussian but Ricean (open symbols). The blue data are the envelope of the laser autocorrelation obtained simultaneously with the Si:P Ramsey fringes using a reference detector. Solid lines are Gaussian fits. Note log-linear scale. See Methods for details of filters, fits, discussion of Ricean noise and so on.

In our experiments, the FELIX laser was tuned to the frequency of the 1*s*–2*p*_±_ transition (see Methods). The beam was separated with a beam splitter into two equal, collinear pump beams (that is, the pulses were split into two, denoted as pulse 1 and pulse 2), each with an independent intensity controller and delay stage (we denote the separation in arrival times as *τ*_12_). The beam was then focused onto the sample, which was liquid at helium temperature. The intensities were adjusted so that both beams 1 and 2 were π/4 pulses (corresponding to the 0.15μJ cm^−2^ peak, in a beam of radius 1 mm and pulse duration 5.7 ps). The photoconductivity signal was measured as a function of *τ*_12_ to give the Ramsey fringes (Figs 1b,c and 2).

Figure 2b shows the electrically detected Ramsey fringes in Si:P (see Methods for a description of the laser, optical apparatus and sample). Short expanded sections contain clear interference fringes in the time domain, but they are much more obvious in the frequency domain as shown by the Fourier transform (FT) computed over each section. The peak at the transition frequency is clearly visible and can still be seen even at 60 ps. Figure 2c shows the amplitude of the fringe envelope as a function of time delay produced with a FT filter (see Methods), along with the noise level, and the equivalent analysis for an autocorrelation of the incident FEL beam taken with a reference detector. The Ramsey interference lasts much longer than the autocorrelation reference. The measured laser spectral width was Δ*f*_L_=0.066±0.001 THz and the autocorrelation fringe duration (blue line from Fig. 2c) is Δ*τ*_L_=17.4±1 ps. The Ramsey fringe duration is Δ*τ*_R_=59±10 ps, (red line in Fig. 2c). In the small-signal PTIS (Fig. 1a) the 1*s*(*A*_1_)–2*p*_±_ line is approximately Gaussian, as expected for an inhomogeneous line, with width Δ*f*_A_=0.028 THz (the spectrum was taken with 0.006 THz resolution).

### Optically detected Ramsey fringes

The Ramsey fringes described so far utilize the simplest possible pulse sequence and demonstrate the electrical detection of quantum superpositions of impurity orbitals in silicon. Although the analysis is straightforward and yields an inhomogeneous dephasing time in good agreement with expectations, it relies on taking account of an inelastic scattering process as well as the Ramsey fringes. It is therefore useful to obtain an independent check of the analysis, and more importantly, to verify that electrical detection in this time-resolved experiment is as straightforward as we posit above. To this end, we devised an all-optical echo detection scheme^15^ that is sensitive only to the coherences produced by the Ramsey fringes and completely eliminates any possible effect of the inelastic scattering component. In this experiment, an initial pump pulse pair (pulses 1 and 2 shown in Fig. 1d for the case that they are π/4 pulses, in phase) forms a coherent superposition of states represented by a rotation of π/2 about the *x* axis, onto the equator of the Bloch sphere. The superposition rotates around the *z* axis at the natural frequency and is allowed to dephase until a third rephasing pulse (pulse 3) gives a rotation about *x* again, this time by π, so that the phase lead/lag is reversed. The superpositions continue to evolve, allowing coherent emission to build up after waiting for a further time *τ*_23_. The echo intensity as a function of *τ*_23_ gives directly the inverse homogeneous phase decoherence rate, *T*_2_^−1^. Inhomogeneous broadening, due to static random fields, is eliminated in the photon echo experiment because the dephasing for which they are responsible is precisely the same before the π pulse and afterwards. In our echo detection of Ramsey fringes, the echo strength is used as a measure of the strength of the coherent superposition produced by the Ramsey pair. The echo exhibits interference fringes with a contrast that reduces as the delay *τ*_12_ approaches the inhomogeneous dephasing time *T*_2_*.

In the optical experiment, the collinear pump beams 1 and 2 produce π/4 pulses as in the previous electrical experiment, but now a rephasing beam (producing a π pulse, beam 3) was also incident on the sample at an angle of about 10° to the pump pulses. The emitted photon echo pulse was then detected at the appropriate angle determined by phase matching by focussing it onto a Ge:Ga cooled detector (see Methods).

**Figure 3:**
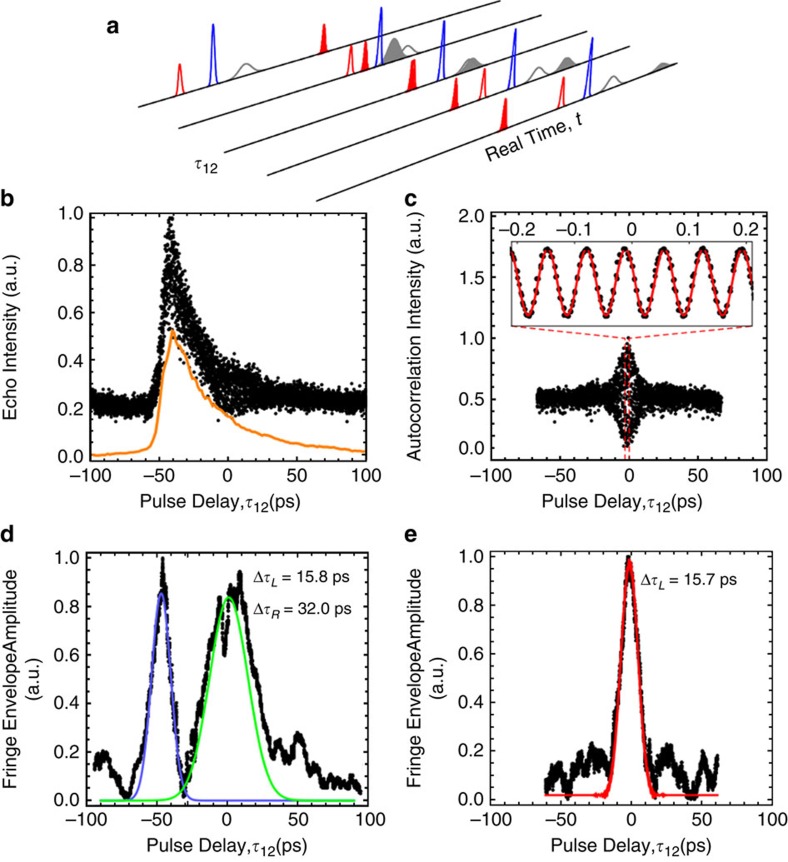
Echo-detected Ramsey fringes. (**a**) Schematic pulse sequence showing the pump pulse 1 (solid red), pump pulse 2 (open red) and rephasing pulse 3 (open blue), and the echoes associated with each pump (grey). (**b**) The detected signal in the echo direction **k**_*e*_ for a laser frequency near resonance with the 1*s*(*A*_1_)→2*p*_0_ transition. The zero of the abscissa (*τ*_12_) occurs when the pumps overlap in time, and the delay between pulses 2 and 3 (*τ*_23_) was fixed at 50 ps. Black points: all three laser pulses unblocked; orange line: pulse 2 blocked showing the two-pulse echo due to pulses 1 and 3. In the black data, there is increased scatter near *τ*_12_=0 (and, less obviously, near *τ*_12_=−50), and this is due to interference fringes, not noise, as shown in other panels. (**c**) The autocorrelation of pulses 1 and 2 with the laser off-resonance (pulse 3 blocked). The inset shows a higher-resolution (step size 0.5 mm) version of the main figure (points) near *τ*_12_=0, with a sine wave fit at the laser frequency (red line). The fringe contrast is ~98%, that is, the minimum is ~1% of maximum. (**d**) The envelope of the fringes in the echo-detected Ramsey fringe data (from **b**), produced with a Fourier transform filter centred on the laser frequency. Two Gaussian fits are shown corresponding to autocorrelation of scatter and the Ramsey fringes (blue and green lines, respectively). (**e**) The envelope of the fringes in the autocorrelation data and a Gaussian fit (red line). See Methods for details of filters, fits, discussion of Ricean noise and so on.

The results (Fig. 3b, black data) for the photon echo signal as a function of *τ*_12_ contain a single-sided exponential, a constant background and large amplitude interference fringes superposed on these features. In the general case of a three-beam experiment, six different echo signals are radiated at different times and directions^23^ because each combination of laser pulses coherently interacts with the wave-like excitations produced by the previous ones. Here, by using collinear pump beams with a rephasing beam at an angle of 10°, and choosing a specific detection direction, we investigate the interaction of only two echo components—one for each of the pumps—that are emitted as a coherent sum. We turn first to the component that produces the fixed background in the signal of Fig. 3b. This is the echo produced by the rephasing pulse with the pump that arrives *τ*_23_ earlier. As *τ*_23_ is fixed, the echo is constant.

The second echo component is due to the rephasing pulse with the other pump, which arrives *τ*_12_+*τ*_23_ earlier. The amplitude is *τ*_12_-dependent due to homogeneous dephasing of the polarization and disappears from view when *τ*_12_+*τ*_23_≤0. It therefore produces the single-sided exponential component rising at *τ*_12_=−*τ*_23_=−50 ps in the signal of Fig. 3b. We recorded this delay-dependent component of the echo signal separately by blocking the other pump (see the orange data in Fig. 3b), and the time constant is 40 ps corresponding to *T*_2_=160 ps (in agreement with ref. 15) where the factor of 4 occurs because the echo intensity is proportional to the square of the amplitude and the time of the echo emission is twice the delay between the pump and rephasing pulses.

The observed Ramsey fringes are produced by interference between the echoes associated with the two pump pulses. Correspondingly, the Ramsey interference signal peaks when the pumps are precisely coincident, that is, for *τ*_12_=0. As well as direct interference between the echoes, there is also interference between scattered light from pulses 1 and 3 (due to slight sample and mirror surface roughness), resulting in interference fringes for photons visible to the detector in the echo direction. This interference peaks when *τ*_12_ is near −50 ps. Because the laser produces FT-limited pulses, the fringe envelope will be the pulse length, while the fringe period will be the inverse laser frequency.

**Figure 4:**
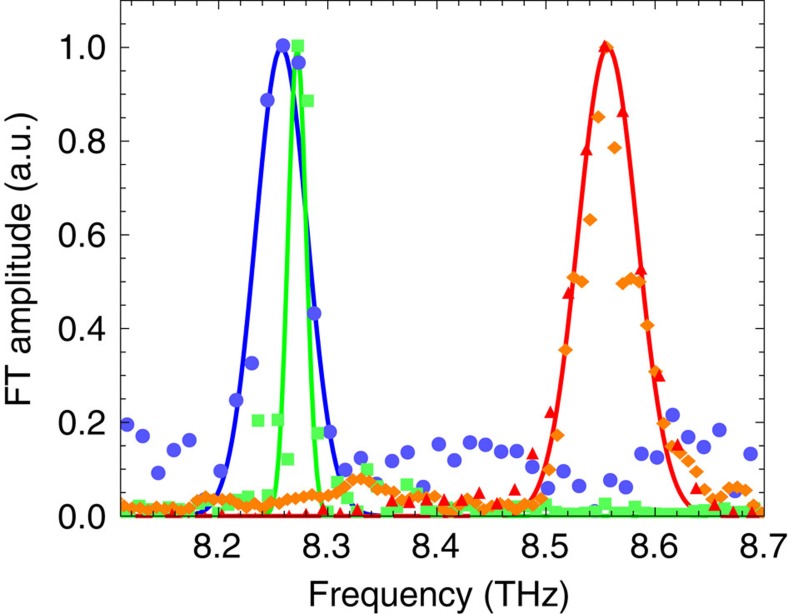
Echo-detected Ramsey fringes in the frequency domain. The green and blue points were obtained by separately applying a Fourier transform (FT) to the echo-detected Ramsey fringes (Fig. 3b) to the right and left (respectively) of *τ*_12_=−30 ps. Red points: the FT of the autocorrelation data (Fig. 3c) and orange points: the measured laser spectrum taken with a monochromator, accumulated simultaneously. The solid lines are not fits of the data points in Fig. 4, they are the FTs of the Gaussian fits to the raw data of Fig. 3, with the colours corresponding.

For quantitative analysis, we have extracted the envelope function for the fringes as described in the Methods section below. Figure 3d shows the outcome, which clearly demonstrates separate peaks due to scatter and to Ramsey fringes, centred at *τ*_12_=−50 and 0 ps, respectively. As expected, the former (Fig. 3d, blue line) is almost identical in width to the autocorrelation (Fig. 3e, red line) whose measured full-width at half-maximum (FWHM) is Δ*τ*_L_=15.7±1.3 ps and corresponds to the measured laser spectral width Δ*f*_L_=0.064±0.001 THz (Fig. 4, orange points). The Ramsey fringes are clearly much broader than the autocorrelation, with measured FWHM Δ*τ*_R_=32.0±1.6 ps (Fig. 3d, green line). The small-signal absorption line (measured by FT infrared spectroscopy with resolution 0.006 THz) had a FWHM Δ*f*_A_=0.046 THz.

In the case of a coherent pulse with a Gaussian envelope, the time–bandwidth product for the linear autocorrelation fringe duration (the FWHM of the envelope amplitude) and the FWHM of the intensity spectrum is Δ*τ*_L_ Δ*f*_L_=4ln2/π=0.88 (independent of any chirp in the pulses). In the electrical and optical experiments Δ*τ*_L_ Δ*f*_L_=1.11±0.07 and 1.00±0.08, respectively, consistent with the fact that pulses show slight departures from Gaussian. When measuring Ramsey interference as a function of the time delay between the pump pulses, the FWHM duration of the fringe amplitude, Δ*τ*_R_, is inversely proportional to the FWHM spectral absorption line width Δ*f*_A_, so it is easy to compare the result with expectation from the frequency domain. Note that one can define a phase coherence time T* or *T*_2_* that is simply proportional to Δ*τ*_R_, but since there are different conventions (for factors of 2 and π) in different research communities, we try to avoid this nomenclature and refer only to the inhomogeneous line width. For a Gaussian inhomogeneous line, the Ramsey fringes follow Δ*τ*_R_ Δ*f*_A_=4ln2/π=0.88 (which is coincidentally the same product as for the autocorrelation fringes). The electrical and optical Ramsey fringe products are Δτ_R_ Δ*f*_A_=1.6±0.4 and 1.5±0.2, respectively, which is partly due to the fact that the fringes are convolved with a laser pulse of finite width, and is also an indication that the absorption line is not a pure Gaussian.

It is useful also to display the results in the frequency rather than the time domain. Figure 4 shows that the elastic-scattering interference peak for *τ*_12_~−50 ps has a width, corresponding to the laser bandwidth, that is substantially larger than the width of the Ramsey interference, corresponding to the narrower width of the 1*s*→2*p* absorption.

Figure 4 shows the FT of the autocorrelation fringes (red) and the comparison with the laser spectrum (orange) off-resonance. All of these measures of the pulse bandwidth, including two time domains (unblocked rephasing pulse for *τ*_12_ near 0 and blocked rephasing pulse for *τ*_12_ near −50 ps) and one frequency domain (measured simultaneously with a grating monochromator) are consistent. We repeated the experiment of Fig. 3 with various pulse durations down to 5.7 ps (corresponding to ~50 cycles at the frequency used here), and while the Ramsey interference is unaffected by the laser pulse duration, the autocorrelation follows the expected time–bandwidth product.

Figure 4 shows that Ramsey spectroscopy is robust and unaffected by the fact that we are pumping slightly off-resonance. It also touches the original intent of Ramsey to see sharp spectral lines in the presence of a broadband source. There are several applications of our results. The first is simply for calibration of pulses to move Bloch vectors to particular locations on the Bloch sphere, useful both for qubits themselves as well as control bits in various quantum computing schemes. The observation of the echo-detected Ramsey fringes themselves entails such control, and the inset of Fig. 3c shows that we are able to achieve mrad settings on the Bloch sphere at 10 THz. Because a phase delay of 2π corresponds to the 36 μm wavelength employed, improvements to our instrument and especially the ability eventually to regulate its settings using piezoelectric transducers with <10 nm resolution should easily allow us to go below a few mrad.

### Photon echo detection method to determine *T*_1_

There are other applications of our three-beam experiments. For example, we can follow the practice of spin resonance spectroscopy and use echo detection to measure spin–lattice relaxation times *T*_1_. This has significant advantages over the more usual pump–probe experiment^13^, namely that there is no possibility of artefacts from induced absorption changes due to any excitations other than the intended two-level atom. Examples of such artefacts include the induced absorption by photoionized electrons (which could remain free or fall into unrelated traps) and the coherent artefact caused by four-wave mixing when the pump and probe beams overlap in time^24^. In this case we use the sequence π-*τ*_12_-π/2-*τ*_23_-π-echo. The first π-pulse is the preparation pulse and the remainder of the sequence produces the read-out echo. If the time delay between the preparation pulse and the echo sequence is very small or very large the polarization is 100% and a full echo is produced in both cases. There is an intermediate delay time for which the populations are equalized, and thus the subsequent pulses produce no echo. For a sequence with general areas and arrival times *A*_1_-*τ*_12_-*A*_2_-*τ*_23_-*A*_3_-echo, the area of the echo in the direction 2**k**_3_–**k**_2_ for short pulses, *τ*_12,23_≥0, and **k**_1_≠**k**_2_≠**k**_3_ may be shown to be


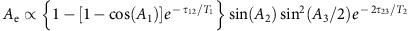


**Figure 5:**
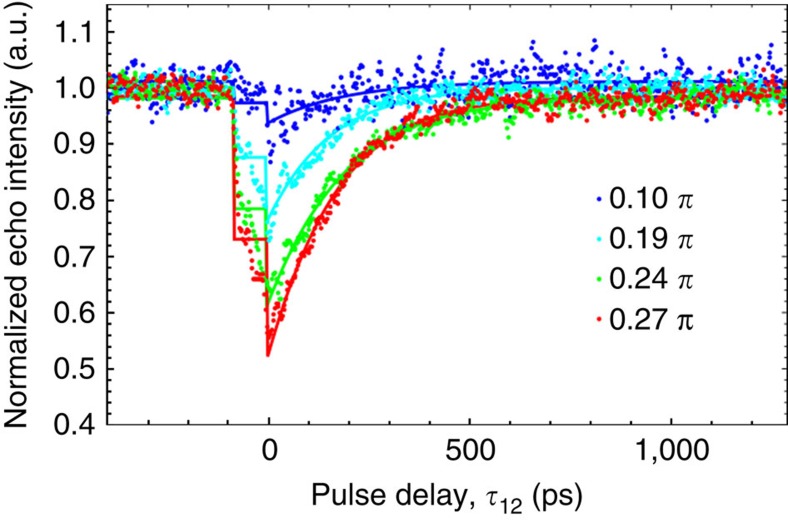
Echo detection of *T*_1_. The echo signal is shown (as points) as a function of the arrival time of the echo sequence relative to the fixed preparation pulse (*τ*_12_). The echo intensity was normalized to its value when the preparation pulse was blocked (*A*_1_=0). At large positive or negative delay the echo is independent of the preparation pulse. At intermediate times the partial relaxation means that the echoes from the excited state and ground state oscillators compete. The solid lines show the theory averaged over the beam profile. NB: for small negative delay −100 ps<*τ*_12_<0, the preparation pulse arrives in the middle of the echo read-out sequence and the response does not follow a simple intuitive picture. The different colours are for different preparation pulse areas *A*_1_.

The first parenthesis is the incoherent population upon arrival of pulse 2, and the remainder is the echo read-out. For experiments on large ensembles such as this one, *A*_1_ is a function of position within the beam spot, and *A*_e_ must be averaged over the beam cross-section. The measured echo intensity is proportional to *A*_e_^2^, and the ordinate of Fig. 5 shows *A*_e_^2^(*A*_1_)/*A*_e_^2^(0), that is, the signal normalized to its value when the preparation beam was blocked. The time constant for the recovery of the echo at large values of *τ*_12_ is just *T*_1_. The lines on Fig. 5 contain no fitting parameters other than *T*_1_, whose value was 180 ps in agreement with previous work^13^.

## Discussion

Using both electrical and optical read-out we have demonstrated the creation and destruction of wavepackets with the Ramsey pulse sequence. The electrical detection of orbital quantum oscillations in silicon is an essential stepping stone for carrying out quantum information experiments in silicon. Ultimately, the role of thermal ionization in the detection may be replaced by tunnelling through the barrier of a single electron transistor, as used for spins. We have shown both electrically and optically that Ramsey spectroscopy is robust and unaffected by the fact that we are pumping slightly off-resonance. We have also developed photon echo spectroscopy techniques not only to observe Ramsey fringes, but also to obtain a more reliable value for *T*_1_ than given by conventional pump–probe spectroscopy. Beyond the obvious applications to basic physics, control and read-out of Rydberg states for the most common impurity in the most common semiconductor will help to implement certain quantum gate architectures for doped silicon^10^.

## Methods

### Time structure of FELIX output

The free-electron laser, FELIX, provides pulses separated by 40 ns, and in bursts of duration 4 μs and repetition frequency 5 Hz. The pulse duration/spectral width control was obtained by detuning of the laser cavity length. This alters the synchronism between the inverse round-trip time of the light pulses in the cavity and the injection frequency of the electron pulses, so that the light pulse duration may be increased without affecting the time–bandwidth product, which is near unity^16^. The pulse energy was controllable up to 10 μJ. A grating spectrometer with a pyroelectric detector array collects a small fraction of the laser light from a hole in one upstream mirror to provide a continually updated spectrum.

### Electrical read-out experiment

**Figure 6:**
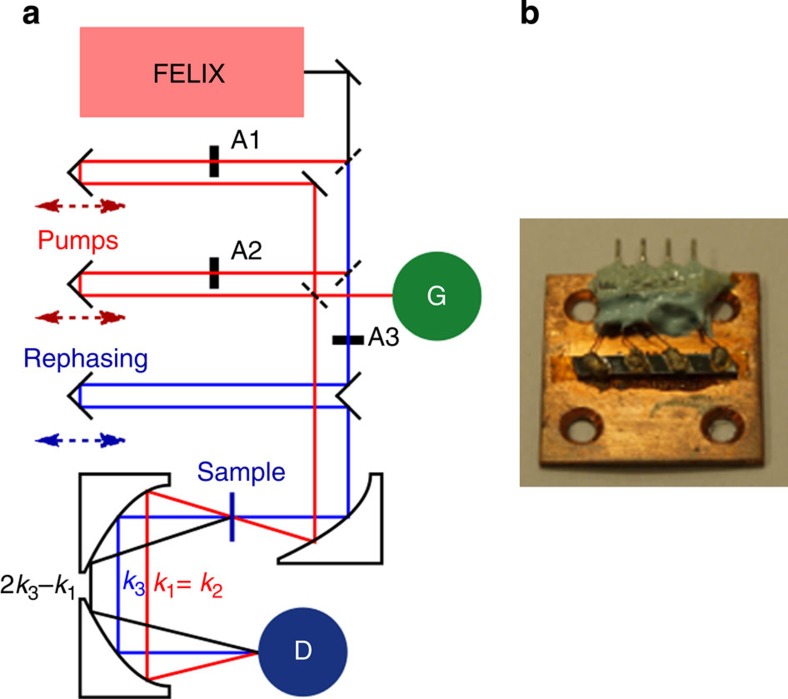
Experimental apparatus. (**a**) The apparatus for the echo detection of Ramsey fringes. A pair of co-linear pumps (red) produce the Ramsey fringes. An echo (black) is induced by the rephasing pulse (blue). A reference detector ‘G’ monitors the laser pulse autocorrelation simultaneously. (**b**) Sample used for electrical experiments. The silicon was contacted with aluminium deposited after an etch using hydrofluoric acid (HF). The sample was kept electrically isolated from the copper plate with kapton tape.

The sample used for electrical read-out was a commercial float zone <110> silicon wafer of 800 μm thickness with a 2 × 10^14^ cm^−3^ phosphorus concentration, with four aluminium contacts in a linear arrangement for four-terminal characterization (Fig. 6). For the photoresponse measurements, a voltage of 12 V was applied across the inner two contacts, which produced a current of 135 μA. PTIS requires slightly elevated temperature to provide sufficient phonons at the excited state ionization energy, but not so much that the ground states start to ionize—the sample was mounted in a continuous flow cryostat with polypropylene windows, in vacuum on the cold finger at 10 K. For small-signal spectroscopy (Fig. 1a), the sample was illuminated with the output of a FTIR interferometer. For Ramsey experiments (Fig. 2), radiation from FELIX was used to illuminate the silicon between the inner contacts. With a beam sizes of ~0.5 mm, around 1.25 × 10^11^ donors were illuminated. The optical arrangement of Fig. 6 was used, but with the detector removed and the rephasing beam blocked.

### Photon echo detection

The sample used for the photon echo detection experiment was a commercially produced float zone <111> silicon wafer of 300 μm thickness with a phosphorus concentration of 6 × 10^14^ cm^−3^. The arrangement of beams is shown in Fig. 6. Calibrated wire mesh attenuators and/or polarizer/analyzer pairs were used to control the intensities. Upon incidence the polarization of the light was always perpendicular to the plane of the bench (S-polarization). The relative arrival times between the pulses were controlled by a simple stepper motor-driven delay stage with a practical resolution of 0.5 μm, easily allowing sub-wavelength phase control. The rephasing pulse had an incidence angle of about 10° to the Ramsey pulse pair, which were co-linear (that is, **k**_1_=**k**_2_). When the rephasing process is complete, the system again exhibits a macroscopic electric dipole moment resulting in a burst of coherent radiation. This photon echo signal is subject to phase-matching conditions, and the echo emission signal was detected in the phase matching **k**_e_=2**k**_3_–**k**_1_ direction. After the sample, the beams were collimated, blocked as required and refocused with off-axis parabolic mirrors onto a helium-cooled Ge:Ga detector. A helium-cooled Ge:Ga photoconductor was also used simultaneously to monitor the autocorrelation signal. In the electrical detection experiment the same arrangement of pumps and reference detector was used (and the rephasing beam was blocked).

For the linear autocorrelation in Fig. 3c, we simply blocked the rephasing beam and detuned the laser away from resonance without altering any alignment, that is, the detector measures scattered light from the pumps (for the inset of Fig. 3c only, to allow accurate estimation of the fringe contrast, we eliminated scatter by removing the sample).

### Data analysis

For the experimental fringe envelopes (Figs 2c,d and 3d,e), the raw fringe data (Figs 2b and 3b,c) were downconverted to d.c. by multiplying the data by exp(*i*2π*f*_L_*τ*_12_), smoothing and then taking the absolute magnitude. This process turns Gaussian noise into low-frequency Ricean noise, which is the reason the noise is smooth, and also why it is positive when the signal is small (giving the impression of a longer tail). For this reason it is crucial to check the noise level by using a similar filter off resonance (as in the examples of Fig. 2c).

For the Gaussian fit to the Fig. 2c autocorrelation data (blue points), the FWHM was a free parameter (along with the height and centre). For the fit to the Ramsey data of Fig. 2c (red points), the sum of two Gaussians was used, but one Gaussian had its parameters fixed equal to the autocorrelation fit, and the other (red line) had its centre fixed equal to the autocorrelation centre with its width and height free.

The Gaussian line-shapes in Figs 3d,e and 4 are all self-consistent: the raw fringe data of Fig. 3b,c were fitted with a sinusoidal fringe pattern having a Gaussian envelope (on top of a background produced by smoothing the data). By fitting only the raw data, instead of the envelopes produced by the FT filtering, we avoid the effects of Ricean noise on the fits (so the fitted envelopes go to zero at large delays as expected). These fits are not shown in Fig. 3b,c (for clarity), but their envelopes are shown in Fig. 3d,e. For the frequency domain (Fig. 4), FTs were applied to the Gaussian fringe fits without further free parameters. In the case of the red and blue curves/points in Fig. 4, the absolute magnitude of the FT (proportional to the power spectrum for an autocorrelation) is shown. In the case of the green curve/points, the magnitude squared of the FT (proportional to the spectral power in a cross-correlation by a short pulse) is shown.

The preparation pulse area *A*_1_ is proportional to 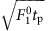 where *t*_p_ is the pulse duration (which is simply proportional to Δ*τ*_L_ for bandwidth-limited pulses) and *F*_1_^0^ is the fluence (energy per unit area) at the centre of the beam spot. Using the dipole moment of *ex*_12_ where *x*_12_=0.28 nm for the 1s(*A*_1_) to 2p_0_ transition^15^, a π-pulse with Δ*τ*_L_=5.7 ps and a Gaussian spatial profile with e-folding radius of 1 mm occurs with *F*_1_^0^=0.3 μJ cm^−2^ (note that this is the fluence outside the sample, and we have taken account of the losses due to reflection, the dielectric constant, polarization and so on).

## Additional information

**How to cite this article:** Litvinenko, K. L. *et al*. Coherent creation and destruction of orbital wavepackets in Si:P with electrical and optical read-out. *Nat. Commun.* 6:6549 doi: 10.1038/ncomms7549 (2015).
