#  Corrigendum: Coherent creation and destruction of orbital wavepackets in Si:P with electrical and optical read-out

**DOI:** 10.1038/ncomms15445

**Published:** 2017-04-20

**Authors:** K. L. Litvinenko, E. T. Bowyer, P. T. Greenland, N. Stavrias, Juerong Li, R. Gwilliam, B. J. Villis, G. Matmon, M. L. Y. Pang, B. Redlich, A. F. G. van der Meer, C. R. Pidgeon, G. Aeppli, B. N. Murdin

Nature Communications 6: Article number: 6549; DOI: 10.1038/ncomms7549 (2015); Published: 03 20 2015; Updated: 04 20 2017

This Article was originally published under a CC BY-NC-ND 4.0 license, but has now been made available under a CC BY 4.0 license. The PDF and HTML versions of the paper have been modified accordingly.

